# Baclofen in alcohol use disorder: An analysis of the data provided by the French “Temporary Recommendation for Use” 2014–2017 cohort

**DOI:** 10.3389/fpsyt.2022.949750

**Published:** 2022-10-12

**Authors:** Renaud de Beaurepaire, Benjamin Rolland

**Affiliations:** ^1^Groupe Hospitalier Paul Guiraud, Villejuif, France; ^2^Academic Department of Addiction Medicine in Lyon, Centre Hospitalier Le Vinatier, Hospices Civils de Lyon, Lyon, France

**Keywords:** alcohol consumption, alcohol craving, high-dose baclofen, baclofen adverse effects, tailored prescription

## Abstract

Alcohol use disorder (AUD) is a devastating illness for which effective treatments are lacking. Studies over the last two decades have shown that baclofen, a GABA-B agonist, could be a promising treatment for AUD. However, the efficacy of baclofen is still controversial, and studies have shown that it may be associated with an excess of hospitalizations and deaths. In March 2014, the French Health Safety Agency granted a “Temporary Recommendation for Use” (TRU) regulating the prescription of baclofen in subjects with AUD. The TRU allowed physicians to prescribe high-dose baclofen (up to 300 mg/d). The doses were adjusted, and tailored to the needs of each patient. Between March 2014 and March 2017, TRU clinical data were collected for a total of 6,939 subjects. The recorded data included information on alcohol consumption, the intensity of alcohol cravings, and adverse events. The present article proposes an analysis of the data provided by the TRU. Subjects for which data were missing regarding baclofen daily dosage, alcohol consumption or craving scores were discarded from the analyses. A cohort of two groups of subjects was analyzed. The first group included all TRU subjects suitable for analyses (5,550 subjects), and the second group included subjects followed for at least 365 days (169 subjects). Comparisons were made between baseline and endpoint of the follow-up period. The results show that a majority of subjects in the whole cohort had received doses of over 80 mg/d. The mean dose of baclofen at the endpoint was >110 mg/d in the second group of subjects. Doses of >80 mg/d were not associated with an increase in adverse events after adjustment for the follow-up duration. In terms of efficacy, comparisons between baseline and endpoint show that baclofen treatment significantly decreased alcohol consumption and alcohol cravings, and significantly increased the number of subjects with null or low-risk alcohol consumption according to WHO criteria.

## Introduction

Alcohol use disorder (AUD) is a devastating illness that affects hundreds of millions of people throughout the world. The disorder comes at a huge medical and societal cost estimated at €120 billion per year in France (1). Medications approved for the treatment of alcohol dependence include acamprosate, naltrexone, nalmefene and disulfiram. These medications are of limited efficacy, and alcohol dependence remains a difficult to treat condition (2). Another medication, baclofen, was recently approved in France by the French Health Safety Agency (ANSM – Agence Nationale de la Sécurité du Médicament) for the treatment of AUD (3). Baclofen is a gamma-aminobutyric acid-B (GABA-B) receptor agonist developed five decades ago for the treatment of spasticity secondary to brain and medulla lesions. For more than 30 years, baclofen was used exclusively by neurologists, and at the beginning of the 2000s, it was shown that baclofen could be effective in the treatment of AUD (4–6). The efficacy of baclofen in AUD has been examined in many observational and randomized controlled trials (RCTs), but still remains controversial because of between-study discrepancies [see references (7, 8) for review]. Between-study discrepancies may be related to many factors (9), one of which is the baclofen dose (low or high) (10, 11).

In France, off-label use of baclofen in the treatment of alcohol dependence increased dramatically following the publication, in 2008, of the book “Le dernier verre” (“The end of my addiction”) (12, 13) by Olivier Ameisen, where the author relates how he cured himself with a high dose of baclofen. The book was a great editorial success, and as a consequence a very large number of alcohol-dependent subjects sought baclofen treatment. In France, the estimated number of subjects taking off-label baclofen for alcohol dependence exceeded 200,000 between 2008 and 2013 (14). To respond to this unexpected situation, the ANSM granted in March 2014 a “Temporary Recommendation for Use” (TRU) regulating the prescription of baclofen in the treatment of alcohol dependence (15). The TRU allowed physicians to prescribe high-dose baclofen (up to 300 mg/d) to AUD subjects under certain conditions. Between March 2014 and March 2017, TRU clinical data were collected for a total of 6,939 subjects. The recorded data included information on daily alcohol consumption, the intensity of alcohol cravings and adverse events. Preliminary results from the TRU study were released by the ANSM in March 2015 (16), and no further analysis of the TRU has been released since. The preliminary TRU results showed a reduction in alcohol consumption and craving in 65 and 74% of the subjects, respectively. The results also showed that at least one adverse event was reported in 14% of the subjects, and a serious adverse event was reported in 1.7%. Another study dedicated to evaluating the security of baclofen was released in 2017 (17). This study showed that, in comparison to other AUD treatments (acamprosate and naltrexone), high doses of baclofen (>80 mg/d), but not low doses (≤80 mg/d), produce an excess of hospitalizations and deaths. The study did not address the question of the efficacy of baclofen. Although the methodology of the study has been critically analyzed (18), the study nevertheless raises the question of the benefits/risks of high-dose baclofen in the treatment of AUD. The TRU database could be a valuable tool for evaluating the efficacy and safety of baclofen, and could provide interesting insights into low-dose/high-dose benefits/risks.

The objective of the present study was to analyze the clinical information provided by the TRU database. The primary objective was to analyze the efficacy and safety of doses of <80 mg (low doses) and doses >80 mg (high doses) for the whole TRU cohort. The aim of the present study was also to compare the effects of baclofen in the TRU with those of studies already published in the literature using high doses of baclofen. Previous studies suitable for comparison with data from the TRU include RCTs and observational studies. RCTs include the BACLAD study (19), the Dutch study (20), the ALPADIR study (21), and the Bacloville study (22). Comparative observational studies include those that, similar to the TRU, used WHO criteria (23) to evaluate the effects of baclofen (10, 11, 24). Previously published RCTs which did not use high doses [including the Garbutt et al. study (25) that was limited to 90 mg/d], and observational studies that used high doses but not WHO criteria as an outcome measure (26–29), were not taken into account in the present article. In terms of efficacy, the results of RCTs were contradictory: BACLAD (19) and Bacloville (22) demonstrated a significant effectiveness of baclofen on alcohol consumption, but no effect on craving, while the ALPADIR study (21) showed a significant effect of baclofen on craving but not on alcohol consumption, and the Dutch study (20) showed no positive effect on both measures. All observational studies using high doses showed positive results on alcohol consumption, none of them evaluated craving. Given that the maximum length of treatment in the published RCTs was 1 year (22), a separate analysis of the efficacy of baclofen in TRU subjects treated for at least 365 days was performed. This analysis was designed to reproduce the conditions of the Bacloville study as much as possible, because the Bacloville study has similarities with the TRU: the use of tailored doses, up to 300 mg/d, and a pragmatic protocol designed to be nearly a real-world study, while other RCTs used fixed doses, lower than 300 mg/day, and more stringent protocol conditions. The efficacy of baclofen in this group of one-year treated TRU subjects was analyzed globally, and by dose (<80 mg and >80 mg). The 80 mg dose was picked as the high vs. low dose cut-off for several reasons: It is the cut-off dose that the ANSM used for baclofen approval in AUD (3); It is the cut-off dose that the ANSM used in their preliminary analyses of the TRU data in 2015 (16); It is the cut-off dose in the study that evaluated the security of baclofen in France in 2017 (17); And the dose that most often separates the high-dose vs. low-dose studies in the literature [see reference (7)].

## Methods

In general, a TRU is a “regulatory framework” granted by the French ANSM allowing physicians to prescribe an off-label medication. The objectives of a TRU are to allow physicians to prescribe a non-approved medication, to collect clinical data, and to implement a pharmacovigilance supervision of prescriptions. The TRU for baclofen was granted for a 3-year period running, from March 2014 to March 2017. Any kind of physician (general practitioner [GP], psychiatrist and other specialists) could include subjects in the cohort. Criteria for inclusion in the baclofen TRU were alcohol dependence, the failure of previous treatment attempts, an absence of significant somatic disorder (kidney and liver diseases, and a history of epilepsy) and mental disorder (severe depression, bipolar disorder, schizophrenia and other psychoses), and an absence of baclofen contra-indication. Medications for alcohol-dependence (acamprosate, naltrexone, nalmefene, disulfirame) were not allowed, these treatments had to be stopped at least 2 weeks before inclusion. Subjects could use any other treatments, including psychosocial treatments, these other treatments were not recorded. Procedures of the baclofen TRU protocol were as follows: tailored prescription of baclofen adjusted to each subject, allowing a maximum daily dose of 300 mg; regular visits to the prescribing physician; and measuring or recording specific variables at each visit. All data were recorded on an online internet portal. The TRU database used for analyses in the present article was exclusively based on the online database. Subjects all gave oral informed consent to participate in the study. The TRU database was acquired by Zentiva Laboratories that performed the statistical analyses and invited the authors to write the present article using the results of these analyses.

### Population

The study pertained to the 6,939 subjects recorded in the TRU online national register. The criteria for inclusion were being diagnosed with AUD, and being started with a treatment of baclofen for AUD, in accordance with the TRU prerequisites, that is, being aged 18 years or older and having no contraindication for baclofen (pregnancy, epilepsy, hypersensitivity or allergy to baclofen or excipients).

### Outcomes

At each recorded visit, which could occur at no predefined time points, prescribers had to note the following variables: (a) average daily consumption, in grams per day (the physicians converted volumes in grams), in the week preceding the visit, (b) average intensity of alcohol cravings in the week preceding the visit (assessed by using a visual analog scale [VAS] graded from zero [no craving] to 10 [permanent and intense craving]), (c) at-risk status using the WHO criteria (that is, high risk for consumption of ≥40 g/d for men and of ≥20 g/d for women), and (d) adverse events: the recording of adverse events was presented in two ways on the Internet portal, first as a list of severe adverse events (fall and fracture, public road accident, severe depression or anxiety, suicidal ideation or behavior, mania, seizures, coma), and, second, as a free-text format space where the physician reported other adverse events. For its part, the ANSM was always informed of the occurrence of the death of a participant. The ANSM decided whether the reported adverse events, or deaths, were related or not to baclofen treatment. Therefore, five categories of adverse events were analyzed: any adverse event, all adverse events related to baclofen treatment, severe adverse events related to baclofen treatment, deaths, deaths related to baclofen treatment.

Subjects included could be either baclofen-free or already taking baclofen. The duration of the follow-up period for each participant ranged from a single visit to the 3-year period of the TRU. The study included 6,939 subjects during the 3-year period. The analyses were separated into two groups:

- Group 1 included all subjects for whom baclofen dosage, alcohol daily consumption and craving scores were available. Once these filters were applied, from a total of 6,939 subjects in the overall population, 1,389 were excluded because of missing information. This left 5,550 subjects for analyses: 1,614 (29%) women and 3,936 (71%) men.

- Group 2 included subjects who had been followed for at least 365 days and were still under baclofen treatment at their first visit after Day 365, for which baclofen dosage, alcohol daily consumption and craving scores were available. Once these filters were applied, a total of 169 subjects (51 [30.18%] women and 118 [69.82%] men) were available for analyses.

Given that the schedules of the visits in the TRU were not regular, but left to the discretion of the physician, the first visit made after 365 days was selected as equivalent to the 12-month visit. This first visit after 365 days took place between exactly 1 year and slightly under 2 years; 50% of the subjects made their visit after Day 399, i.e., approximately 1 month after 1 year, and 75% made their visit after Day 429, i.e., within 2 months of the one-year period. All subjects from group 2 also belong to group 1, but there was actually no comparison between groups. Analyses were essentially sensitivity analyses on both groups. To collect further information regarding the baclofen dosage (low vs. high), another series of analyses was performed in a subgroup of Group 2. This subgroup was limited to subjects who were treated at any time with doses over 80 mg/d (147 subjects).

### Statistics

Descriptive results providing effectiveness measures of the treatment were obtained by calculating the differences between baseline and endpoint scores (any treatment duration in Group 1, at least 365 days of treatment in Group 2) for daily alcohol consumption, alcohol cravings, and at-risk status using the WHO criteria (23). We compared the above scores for the ≤80 mg and >80 mg groups. Two-way ANOVA calculations were performed for daily alcohol consumption, craving scores and at-risk status in the groups and according to the treatment dose reached: ≤80 mg and >80 mg. Two treatment dimension features were analyzed during the treatment periods, with one studied according to the last recorded dose and one studied according to the maximum recorded dose. *P-*values were obtained from Wilcoxon Signed Rank Test and Chi-square test results. The five categories of adverse events were analyzed by posology groups (≤80 mg and >80 mg) in Group 1, and the Incidence of the number of adverse events was adjusted to the follow-up duration (adverse events/subject-year).

## Results

### Group 1

Among the 5,550 subjects, the last recorded dose was ≤80 mg/d in 2,740 subjects (49.37%), and >80 mg/d in 2,810 subjects (50.63%) (Table 1A). The maximum recorded dose was ≤80 mg/d in 2,296 subjects (41.37%), and >80 mg/d in 3,254 subjects (58.63%) (Table 1B). Therefore, a majority of subjects had a last dose and a maximum dose of over 80 mg/d. The average last recorded dose was 48.2 mg in subjects taking ≤80 mg/d and 140.6 mg in subjects taking >80 mg/d (Table 2A). The average maximum recorded dose was 50.8 mg in subjects taking ≤80 mg/d and 146.4 mg in subjects taking >80 mg/d (Table 2B).

**Table 1A:** Percentage of subjects with last recorded dose ≤ or > to 80 mg/day.

**Last recorded dose**	***N* (%)**
≤80 mg/day	2,740 (49.37%)
>80 mg/day	2,810 (50.63%)

**Table 1B:** Percentage of subjects with maximum recorded dose ≤ or > to 80 mg/day.

**Maximum recorded dose**	***N* (%)**
≤80 mg/day	2,296 (41.37%)
>80 mg/day	3,254 (58.63%)

**Table 2A:** Last recorded dose for the overall population.

	**≤80 mg/day**	**>80 mg/day**
*N*	2,740	2,810
Mean (SD)	48.2 (20.46)	140.6 (51.02)
Median	50	120
Percentiles 25, 75	30, 60	100, 160
Min, max	0, 80	120

**Table 2B:** Maximum recorded dose for the overall population.

	**≤80 mg/day**	**>80 mg/day**
N	2,296	3,254
Mean (SD)	50.8 (19.60)	146.4 (55.67)
Median	60	120
Percentiles 25, 75	30, 60	100, 180
Min, max	0, 80	85, 600

The average alcohol consumption according to the last recorded dose was 69.9 g/d at baseline and 36.6 g/d at the last visit for subjects taking ≤80 mg/d (*p* < 0.0001) and 74.2 g/d at baseline and 38.6 g/d at the last visit for subjects taking >80 mg/d (*p* < 0.0001) (Table 3A). There were no significant differences between the two dose groups (*p* = 0.45). Alcohol consumption according to the maximum dose was 70.5 g/d at baseline and 39.1 g/d at the last visit for subjects taking ≤80 mg/d (*p* < 0.0001) and 73.1 g/d at baseline and 36.6 g/d at the last visit for subjects taking >80 mg/d (*p* < 0.0001) (Table 3B). There were no significant differences between the 2 dose groups (*p* = 0.1).

**Table 3A:** Alcohol consumption for the overall population – last recorded dose.

	**Last recorded dose**		
**Baseline**	**≤80 mg/day**	**>80 mg/day**	
*N*	2,077	2,156	
Mean (SD)	69.9 (89.04)	74.2 (91.71)	
Median	50	50	
Percentiles 25, 75	0, 100	0, 100	
Min, max	0, 900	0,840	
			*P =* 0.45
Last visit			
*N*	2,682	2,738	
Mean (SD)	36.6 (64.13)	38.6 (60.42)	
Median	10	18	
Percentiles 25, 75	0, 50	0, 50	
Min, Max	0, 750	0,600	
	*P* < 0.0001	*P* < 0.0001	

**Table 3B:** Alcohol consumption for the overall population – maximum recorded dose.

	**Maximum recorded dose**		
**Baseline**	**≤80 mg/day**	**>80 mg/day**	
*N*	1,642	2,591	
Mean (SD)	70.5 (88.37)	73.1 (91.71)	
Median	50		
Percentiles 25, 75	0, 100	0, 100	
Min, max	0, 750	0,900	
			*P =* 0.1
Last visit			
*N*	2,242	3,178	
Mean (SD)	39.1 (66.91)	36.6 (58.79)	
Median	10	10	
Percentiles 25, 75	0, 50	0, 50	
Min, max	0, 750	0,600	
	*P* < 0.0001	*P* < 0.0001	

The average alcohol craving scores according to the last recorded dose were 5.4 at baseline and 3.5 at the last visit for subjects taking ≤80 mg/d (*p* < 0.0001) and 5.3 at baseline and 3.6 at the last visit for subjects taking >80 mg/d (*p* < 0.0001) (Table 4A). There were no significant differences between the two dose groups (*p* = 0.58). Alcohol craving scores according to the maximum dose were 5.5 at baseline and 3.7 at the last visit for subjects taking ≤80 mg/d (*p* < 0.0001) and 5.3 at baseline and 3.4 at the last visit for subjects taking >80 mg/d (*p* < 0.0001) (Table 4B). There were no significant differences between the two dose groups (*p* = 0.78).

**Table 4A:** Alcohol craving for the overall population – last recorded dose.

	**Last recorded dose**		
**Baseline**	**≤80 mg/day**	**>80 mg/day**	
*N*	2,087	2,162	
Mean (SD)	5.4 (3.02)	5.3 (2.92)	
Median	6		
Percentiles 25, 75	3.8	3.8	
Min, max	0,10	0,10	
			*P =* 0.58
Last visit			
*N*	2,673	2,746	
Mean (SD)	3.5 (2.86)	3.6 (2.67)	
Median	3		
Percentiles 25, 75	1.6	1.5	
Min, max	0,10	0,10	
	*P* < 0.0001	*P* < 0.0001	

**Table 4B:** Alcohol craving for the overall population – maximum recorded dose.

	**Maximum recorded dose**		
**Baseline**	**≤80 mg/day**	**>80 mg/day**	
*N*	1,649	2,600	
Mean (SD)	5.5 (2.97)	5.3 (2.97)	
Median	6		
Percentiles 25, 75	3.8	3.8	
Min, max	0,10	0,10	
			*P =* 0.78
Last visit			
*N*	2,238	3,181	
Mean (SD)	3.7 (2.87)	3.4 (2.69)	
Median	3		
Percentiles 25, 75	1.6	1.5	
Min, max	0,10	0,10	
	*P* < 0.0001	*P* < 0.0001	

The percentage of subjects with null or low-risk alcohol consumption according to the last recorded dose was 46.53 at baseline and 68.58% at the last visit for subjects taking ≤80 mg/d (*p* < 0.0001) and 46.98 at baseline and 67.76% at the last visit for subjects taking >80 mg/d (*p* < 0.0001) (Table 5A). There were no significant differences between the two dose groups (*p* = 0.77 at baseline and *p* = 0.51 at the last visit). The percentage of subjects with null or low-risk alcohol consumption according to the maximum dose was 45.25 at baseline and 66.59% at the last visit for subjects taking ≤80 mg/d (*p* < 0.0001) and 47.72 at baseline and 69.27% at the last visit for subjects taking >80 mg/d (*p* < 0.0001) (Table 5B). There were no significant differences between the two dose groups at baseline (*p* = 0.11), but the difference was significant at the last visit (*p* = 0.035), possibly demonstrating a better effectiveness of high-dose baclofen treatment. However, the duration of treatment was different according to the dose: 271.5 days according to the last recorded dose and 225.4 days according to the maximal recorded dose in the ≤80 mg/d group vs. 389.9 days according to the last recorded dose and 406.5 days according to the maximal recorded dose in the >80 mg/d group (Tables 5A,B). There was, therefore, a decrease in the percentage of alcohol dependent subjects according to WHO criteria in the two groups, with the high-dose treatment potentially being superior. The fact that a substantial proportion of subjects were at null or low risk at baseline is addressed in the discussion section.

**Table 5A:** Percentage of subjects with null or low-risk alcohol consumption for the last recorded dose.

	**Last recorded dose**			
**Dose group**	**Baseline (%)**	**Last visit (%)**	***P-*value**	**Average follow-up time (days)**
≤80 mg/day	2,106 (46.53%)	2,740 (68.58%)	<0.0001	271.5
>80 mg/day	2,184 (46.98%)	2,810 (67.76%)	<0.0001	389.9
*P*-value	0.77		0.51	

**Table 5B:** Percentage of subjects with null or low-risk alcohol consumption for the maximum recorded dose.

	**Maximum recorded dose**			
**Dose group**	**Baseline (%)**	**Last visit (%)**	***P-*value**	**Average follow-up time (days)**
≤80 mg/day	1,662 (45.25%)	2,296 (66.59%)	<0.0001	225.4
>80 mg/day	2,628 (47.72%)	3,254 (69.27%)	<0.0001	406.5
*P*-value	0.11		0.035	

Adverse events were analyzed according to the posology groups. In the ≤80 mg/d group, 14.98% of subjects reported an adverse event, while 9.89% had an adverse event attributed to baclofen, 1.87% had a severe adverse event, 0.13% died, and in 0.04% of the cases of death, the death was attributed to baclofen. In the >80 mg/d group, the same percentages were 23.26, 15.46, 3.75, 0.15 and 0.09%, respectively (Table 6A). When adjusted to the duration of treatment, incidences of adverse events were 0.472, 0.304, 0.049, 0.002 and 0.001 in the ≤80 mg/d group, and 0.489, 0.316, 0.055, 0.002 and 0.001 in the >80 mg/d group, respectively (Table 6B). Therefore, after adjusting for the follow-up duration, the incidence of the different types of adverse events was similar between the two groups.

**Table 6A:** Adverse events by posology groups.

**AE type**	**Events**	**Events**	**Subjects**	**Subjects**
	**≤80 mg/day**	**>80 mg/day**	**≤80 mg/day**	**>80 mg/day**
Any AE	662	1,732	344 (14.98%)	757 (23.26%)
Related AE	426	1,120	227 (9.89%)	503 (15.46%)
Severe AE	69	196	43 (1.87%)	122 (3.75%)
Deaths	3	5	3 (0.13%)	5 (0.15%)
Related deaths	1	3	1 (0.04%)	3 (0.09%)

**Table 6B:** Incidence of the number of adverse events/subject-year.

**AE type**	**Events**	**Events**
	**≤80 mg/day**	**>80 mg/day**
Any AE	0.472	0.489
Related AE	0.304	0.316
Severe AE	0.049	0.055
Deaths	0.002	0.002
Related deaths	0.001	0.001

Comparisons between women and men showed no significant gender difference for any of the variables (data not shown).

### Group 2

Daily mean alcohol consumption significantly decreased from 88.4 g/d at baseline to 24.3 g/d at their first visit after Day 365 (*p* < 0.0001) (Table 7). The craving score significantly decreased from 6.8 at baseline to 2.7 at their first visit after Day 365 (*p* < 0.0001) (Table 8). Overall, 28.4% of the group had null or low-risk consumption (WHO criteria) at baseline and 75.15% did so at their first visit after Day 365, and the difference was statistically significant (*p* < 0.0001) (Table 9). The significant decrease in consumption and craving and the significant increase in null or low-risk consumption (always *p* < 0.0001) were similar in men and women (data not shown). Therefore, globally, baclofen treatment in this group of subjects significantly decreased alcohol consumption and craving and improved recovery according to WHO criteria. The mean daily dose of baclofen at their first visit after Day 365 was 110.3 mg/d for the whole group (Table 10) (111.2 mg/d in women and 109.9 mg/d in men, a non-significant difference [*p* < 0.65]).

**Table 7:** Alcohol consumption in Group 2.

	**Baseline**	**12 months**	***P-*value**
*N*	168	168	
Mean (SD)	88.4 (78.82)	24.3 (45.97)	
Median	70	4	<0.0001
Percentiles 25, 75	30, 130	0, 40	
Min, max	0, 370	0, 330	

**Table 8:** Craving for alcohol in Group 2.

	**Baseline**	**12 months**	***P-*value**
N	168	168	
Mean (SD)	6.8 (2.10)	2.7 (2.48)	
Median	7	3	<0.0001
Percentiles 25, 75	5, 8	0, 5	
Min, max	0, 10	0, 9	

**Table 9:** Percentage of subjects with a null or low-risk consumption in Group 2.

**Overall**	**Baseline (%)**	**12 months (%)**	***P-*value**
	28.40%	75.15%	<0.0001

**Table 10:** Daily dose for the global population in Group 2 (first visit after 1 year of treatment).

	**Value (mg/day)**
*N*	169
Mean (SD)	110.3 (67.17)
Median	90
Percentiles 25, 75	60, 150
Min, max	10, 400

Analyses of the subgroup of subjects treated at any time with doses >80 mg/d show that this group included 140 subjects (86.98% of Group 2), 41 women (29.28%) and 99 men (70.71%), with an average follow-up of 409.8 days. Daily mean consumption significantly decreased from 95.6 g/d at baseline to 25.0 g/d at their first visit after Day 365 (*p* < 0.0001) (Table 11). The craving score significantly decreased from 6.9 at baseline to 2.7 at their first visit after Day 365 (*p* < 0.0001) (Table 12). The proportion of subjects with null or low-risk alcohol consumption significantly increased from 27.14 at baseline to 74.29% at their first visit after Day 365 (*p* < 0.0001) (Table 13). The mean daily dose at 12 months was 123.9 mg/d (Table 14). The significant decrease in consumption and craving, and the significant increase in null or low-risk consumption were similar in men and women (always *p* < 0.0001) (data not shown).

**Table 11:** Alcohol consumption in Group 2 subjects who were administered more >80 mg/day at any timepoint.

	**Baseline**	**12 months**	***P-*value**
*N*	139	139	
Mean (SD)	95.6 (82.95)	25.0 (45.83)	<0.0001
Median	75	3	
Percentiles 25, 75	30, 150	0, 40	
Min, max	0, 370	0, 330	

**Table 12:** Craving scores in Group 2 subjects who were administered more >80 mg/day at any timepoint.

	**Baseline**	**12 months**	***P-*value**
N	140	139	
Mean (SD)	6.9 (2.09)	2.7 (2.51)	<0.0001
Median	7	2	
Percentiles 25, 75	5, 8	0, 5	
Min, max	0, 10	0, 9	

**Table 13:** Percentage of subjects with a null or low-risk consumption in Group 2 subjects who were administered >80 mg/day at any timepoint.

**Overall**	**Baseline (%)**	**12 months (%)**	***P-*value**
	27.14%	74.29%	<0.0001

**Table 14:** Daily dose at the first visit after one year of treatment in Group 2 subjects who were administered >80 mg/day at any timepoint.

	**Value (mg/day)**
*N*	140
Mean (SD)	123.9 (65.58)
Median	120
Percentiles 25, 75	80, 150
Min, max	20, 400

## Discussion

The present analyses of the TRU database show that, in clinical practice, using tailored doses of baclofen in AUD subjects was associated with significant decreases in alcohol consumption, alcohol cravings, and high-risk consumption status according to WHO criteria. The findings were similar for both group doses. Even though these results are not controlled, which does not allow us to affirm that the effect is due to baclofen, they suggest that the global treatment allows us to significantly support drinking reduction in subjects treated with baclofen for AUD. The results also show that a majority of subjects took doses over 80 mg/d during the period observed. Doses of over 80 mg/d were not associated with a substantial increase in serious adverse events. The individualization of Group 2 subjects in the present study makes it particularly suitable for comparison to the Bacloville study, a pragmatic prospective study where outpatients were given tailored doses of baclofen up to 300 mg/d, and were followed for 1 year (22). The analysis of this subgroup of subjects showed that a large majority of subjects treated for a long period of time during the TRU had a high dose of baclofen (86.98% of the whole group) and that this high-dose treatment was very effective in terms of consumption and craving and increased the number of null or low-risk alcohol consumption cases according to WHO criteria.

The follow-up period was relatively short (<1 year) for a large majority of the subjects since only 169 of them could be reliably followed for at least 1 year. A loss of participants during the follow-up can be explained in several ways: A substantial loss of participants is common in clinical trials testing baclofen in AUD (40% discontinuation rate in the ALPADIR study, 32% in the Bacloville study). Adverse effects are a frequent cause of early treatment cessation. A number of subjects are satisfied with a period of treatment limited to a few months (sometimes, baclofen is effective after a short period of treatment). The TRU was a real-world study and some participants may have changed of practitioner during the follow-up (GPs often take over when treatment has stabilized). Baclofen is not a well-established treatment of AUD and, during the 2014-2017 period, many negative messages were delivered in the media and social networks that could have discourage participants to continue their treatment. Moreover, many subjects were already treated by baclofen when included into the cohort, and therefore had a much longer period of treatment than that recorded in the study.

In the Group 2 analysis set, the daily consumption of alcohol dropped from 88.4 g/d at baseline to 22.3 g/d at their first visit after Day 365. The 88.4 g/d consumption at baseline was lower than the 129 g/d consumption in the Bacloville study (22). However, given that 28.40% of subjects in the TRU were in detox at baseline, the pretreatment alcohol consumption of subjects before they started baclofen was likely higher than 88.4 g/d, presumably in the range of that of the Bacloville study (22). At the endpoint, the 24.3 g/d consumption in the TRU cohort did not differ much from the 34 g/d consumption in the Bacloville study (22). However, in the Bacloville study, daily alcohol consumption at 12 months was estimated by multiple imputation for missing data, a method that cannot be compared to the TRU data analyses, as the data were not collected according to a fixed monthly schedule as in the Bacloville study. The consumption at the endpoint in Bacloville (22) was 45 g/d in the placebo group, and the difference between the baseline and endpoint was not significant. In the ALPADIR study (21), the mean baseline and endpoint alcohol consumption in the treated group were 93.6 g/d and 38.5 g/d, respectively, a difference not significantly different from the placebo group. Mean baseline consumption of alcohol was higher in the BACLAD study (19) at 191.6 g/d, but the endpoint consumption is not given in the article. Endpoint alcohol consumption was not measured in the Dutch RCT (20) or observational studies (10, 11, 24).

The craving score for alcohol, evaluated by a VAS, decreased significantly between the baseline and endpoint from 6.8 to 2.7. The VAS evaluation of craving was used in the Bacloville (22) and BACLAD (19) studies and not in other studies. The VAS score at the endpoint is not given in the Bacloville study. No significant effect of treatment on the VAS was found in the BACLAD study (19). The Obsessive-Compulsive Drinking Scale (OCDS) is another method for evaluating craving. It was used in the four RCTs, and comparisons between the baseline and endpoint were made in the four studies. No significant effect was found in the studies, except in the ALPADIR (21) study where a significant decrease was found between the baseline and endpoint. The craving score was not measured in the observational studies (10, 11, 24). Although the method of evaluation was different, the ALPADIR (21) study is the only study in accordance with the TRU regarding the measurement of alcohol cravings.

The proportion of subjects with null or low-risk consumption increased from 28.40 to 75.15% between the two time points, which is a 46.5% increase. The reasons why 28.40% of the subjects were at null or low risk consumption at baseline were, first, that certain prescribers required their subjects to be detoxified from alcohol before starting baclofen, a common practice in alcohol dependence treatment in France, and, second, that a number of subjects included in the TRU cohort were already treated with baclofen, with baclofen treatment being already effective in many of them. The existence of 28.40% of subjects rated as null or low-risk at baseline strengthens the significant increase in subjects who went from being at risk at baseline to having a null or low-risk case at endpoint. The percentages of abstinence or low-risk subjects at endpoint is 57% in the treatment group vs. 36% in the placebo group (a statistically significant difference) in the Bacloville study (22), and respectively 11.9 and 10.5% (a non-significant difference) in the ALPADIR study (21). In the BACLAD study (19), 68.2% of subjects were abstinent during the treatment phase, but it is not mentioned whether this was set as the endpoint percentage. This percentage is in accordance with the range in the TRU. Percentages in high-and low-risk subjects at the baseline and endpoint are not mentioned in the Dutch study (20). The 46.5% increase in subjects with null or low-risk consumption between the two time points in the TRU is in the range of that of observational studies, where the number of subjects at null or low risk at 1 year was 80% in Rigal et al. study (10) and 62% in de Beaurepaire study (11), and 63% at 3 years (the number at 1 year is not given) in the Pinot et al. study (24).

In our study, analyses did not integrate time-dependent variables, and the dose considered was merely the maximum dose reached throughout the follow-up duration, which could be very variable between subjects. The mean maximum dose of baclofen at the endpoint was 110.3 mg/d. The mean dose of baclofen at the endpoint is not given in RCTs but is available in two observational studies as 129 mg/d in the Rigal et al. study (10) and 100 mg/d in the Pinot et al. study (24). These doses are very similar to those of the TRU register. On the other hand, the mean maximum doses prescribed were mentioned in all RCTs and observational studies. Among RCTs, the levels are: 191.3 mg/d for BACLAD (19), 153 mg/d for ALPADIR (21), 93.6 mg/d for the Dutch study (20), and 180 mg/d for Bacloville (22). Among observational studies, the levels are: 145/d for the Rigal et al. study (10), 147/d for the de Beaurepaire study (11), 211/d for the Pinot et al. study (24). These results indicate that in studies demonstrating the effectiveness of baclofen (10, 11, 19, 22, 23), the mean maximum dose was always elevated. This supports the use of tailored but possibly high doses of baclofen in AUD, when the tolerability is correct, as high doses can also contribute to the triggering of sedation (30).

The present study has several limitations. The first is the amount of missing data: In Group 1, 1,389 (20%) of the 6,939 TRU subjects were discarded from the analyses due to missing data. Another limitation is that in Group 2, the visit considered as the 12-month visit for TRU subjects varied from 1 year to nearly 1 years for some subjects. However, this visit occurred within 14 months for the vast majority of subjects. There may also have been bias in the selection and follow-up of participants because physicians who included subjects in the TRU cohort were often very interested in the use of baclofen in AUD and therefore particularly attentive to the management and prevention of severe adverse effects; this could in part explain the discrepancies between the results of the present study and those of the study sponsored by the ANSM (17) in terms of severe adverse events. The comparison of the TRU with RCTs is also problematic, since the methodologies of the TRU and RCTs are very different, possibly too different to allow reliable comparisons.

In conclusion, the present analysis of the data from the TRU cohort shows that high doses of baclofen were commonly used in France for the treatment of AUD subjects in the period of time studied. A majority of subjects in the cohort took doses of over 80 mg/d. Doses of over 80 mg/d were not associated with a dramatic increase in adverse events or serious adverse events. In terms of effectiveness, the results show that tailored doses of baclofen in AUD subjects were associated with a significant decrease in the average level of alcohol consumption, in the average intensity of alcohol cravings, and in the proportions of subjects with high-risk consumption according to WHO criteria. Globally, the TRU real-life study shows that when baclofen doses are adapted to each subject, they can be very useful in the treatment of AUD.

## Data availability statement

The raw data supporting the conclusions of this article will be made available by the authors, without undue reservation.

## Ethics statement

Ethical review and approval was not required for the study on human participants in accordance with the Local Legislation and Institutional Requirements. Written informed consent for participation was not required for this study in accordance with the National Legislation and the Institutional Requirements.

## Author contributions

Both authors listed have made a substantial, direct, and intellectual contribution to the work and approved it for publication.

## Conflict of interest

The authors declare that the research was conducted in the absence of any commercial or financial relationships that could be construed as a potential conflict of interest.

## Publisher's note

All claims expressed in this article are solely those of the authors and do not necessarily represent those of their affiliated organizations, or those of the publisher, the editors and the reviewers. Any product that may be evaluated in this article, or claim that may be made by its manufacturer, is not guaranteed or endorsed by the publisher.

## References

[1] Kopp P. Social Cost of Substance use in France [Le coût social des drogues en France]. OFDT. (2015). Available online at: https://www.ofdt.fr/publications/collections/notes/le-cout-social-des-drogues-en-france/ (accessed October 1, 2022).

[2] Anderson P Møller L Galea G. Alcohol in the European Union: Consumption, Harm Policy Approaches. Copenhagen: World Health Organization Regional Office for Europe. (2012). Available online at: https://apps.who.int/iris/handle/10665/107301 (accessed October 1, 2022).

[3] Agence nationale de sécurité du médicament et des produits de santé (ANSM). L'ANSM octroie une autorisation de mise sur le marché pour une utilisation du bacloféne dans l'alcoolo-dépendance – Statement October 2018 (2018). Available online at: https://ansm.sante.fr/actualites/lansm-octroie-une-autorisation-de-mise-sur-le-marche-pour-une-utilisation-du-baclofene-dans-lalcoolo-dependance-communique (accessed October 1, 2022).

[4] Addolorato G Caputo F Capristo E Colombo G Gessa GL Gasbarrini G. Ability of baclofen in reducing alcohol craving and intake: II – Preliminary clinical evidence. Alcohol Clin Exp Res. (2000) 24:67–71. 10.1111/j.1530-0277.2000.tb04555.x 10656195

[5] Addolorato G Caputo F Capristo E Domenicali M Bernardi M Janiri L. (2002). Baclofen efficacy in reducing alcohol craving and intake: a preliminary double- blind randomized controlled study. Alcohol Alcohol. (2000) 37:504–8. 10.1093/alcalc/37.5.504 12217947

[6] Ameisen O. Complete and prolonged suppression of symptoms and consequences of alcohol-dependence using high-dose baclofen: a self-case report of a physician. Alcohol Alcohol. (2005) 40:147–50. 10.1093/alcalc/agh130 15596425

[7] de Beaurepaire R Sinclair JMA Heydtmann M Addolorato G. The use of baclofen as a treatment for alcohol use disorder: a clinical practice perspective. Front Psychiatry. (2019) 9:708. 10.3389/fpsyt.2018.00708 30662411 PMC6328471

[8] Agabio R Sinclair JM Addolorato G Aubin HJ Beraha EM Caputo F. Baclofen for the treatment of alcohol use disorder: the Cagliari Statement. Lancet Psychiatry. (2018) 5:957–60. 10.1016/S2215-0366(18)30303-1 30413394 PMC12440138

[9] Leggio L Garbutt JC Addolorato G. Effectiveness and safety of baclofen in the treatment of alcohol dependent patients. CNS Neurol Disord Drug Targets. (2010) 9:33–44. 10.2174/187152710790966614 20201813

[10] Rigal L Alexandre-Dubroeucq C de Beaurepaire R Le Jeunne C Jaury P. Abstinence and ‘low risk' consumption one year after the initiation of high-dose baclofen: a retrospective study among ‘high risk' drinkers. Alcohol Alcohol. (2012) 47:439–42. 10.1093/alcalc/ags028 22434664

[11] de Beaurepaire R. Suppression of alcohol dependence using baclofen: a 2-year observational study of 100 patients. Front Psychiatry. (2012) 3:103. 10.3389/fpsyt.2012.00103 23316172 PMC3540966

[12] Ameisen O. Le Dernier Verre. Paris: Denoël. (2008).

[13] Ameisen O. The End of My Addiction. New York: S Crichton Books. (2008).

[14] Chaignot C Weill A Ricordeau P Alla F. Use in France of baclofen for alcohol dependence from 2007 to 2013: cohort study based on the databases SNIIRAM and PMSI. Thérapie. (2015) 70:443–53. 10.2515/therapie/2015027 26423143

[15] Agence nationale de sécurité du médicament et des produits de santé (ANSM). Une recommandation temporaire d'utilisation (RTU) est accordée pour le bacloféne – Statement March 2014 (2014). Available online at: https://ansm.sante.fr/actualites/une-recommandation-temporaire-dutilisation-rtu-est-accordee-pour-le-baclofene (accessed October 1, 2022).

[16] Agence nationale de sécurité du médicament et des produits de santé (ANSM). RTU baclofène: Premières données collectées et rappels sur les modalités de prescription – Statement October 2015 (2015). Available online at: https://ansm.sante.fr/actualites/rtu-baclofene-premieres-donnees-collectees-et-rappels-sur-les-modalites-de-prescription (accessed October 1, 2022).

[17] Agence nationale de sécurité du médicament et des produits de santé (ANSM). Le Baclofène en vie réelle en France entre 2009 et 2015. Usages, persistance et sécurité, et comparaison aux traitements des problèmes d'alcool ayant une autorisation de mise sur le marché. Statement June 2017 (2017). Available online at: https://assurance-maladie.ameli.fr/etudes-et-donnees/2017-baclofene-2009-2015 (accessed October 1, 2022).

[18] de Beaurepaire R Benyamina A Granger B Hill C Sicard D Jaury J. Sécurité du baclofène: l'étrange appréciation de l'Agence française du medicament. Psychiat Sci Hum. (2018) 3:37–53. 10.3917/psn.163.0037

[19] Müller CA Geisel O Pelz P Higl V Krüger J Stickel A. High-dose baclofen for the treatment of alcohol dependence (BACLAD study): a randomized, placebo-controlled trial. Eur Neuropsychopharmacol. (2015) 25:1167–177. 10.1016/j.euroneuro.2015.04.002 26048580

[20] Beraha EM Salemink E Goudriaan AE Bakker A de Jong D Smits N. Efficacy and safety of high-dose baclofen for the treatment of alcohol dependence: a multicentre, randomised, double-blind controlled trial. Eur Neuropsychopharmacol. (2016) 26:1950–9. 10.1016/j.euroneuro.2016.10.006 27842939

[21] Reynaud M Aubin HJ Trinquet F Zakine B Dano C Dematteis M. A randomized, placebo-controlled study of high-dose baclofen in alcohol-dependent patients – The ALPADIR Study. Alcohol Alcohol. (2017) 52:439–46. 10.1093/alcalc/agx030 28525555

[22] Rigal L Sidorkiewicz S Tréluyer JM Perrodeau E Le Jeunne C Porcher R. Titrated baclofen for high-risk alcohol consumption: a randomized placebo-controlled trial in out-patients with 1-year follow-up. Addiction. (2020) 115:1265–76. 10.1111/add.14927 31833590

[23] World Health Organization. International Guide for Monitoring Alcohol Consumption and Related Harm. Geneva: Department of Mental Health, and Substance Dependence, Noncommunicable Diseases, and Mental Health Cluster, World Health Organization (2000). Available online at: https://apps.who.int/iris/handle/10665/66529 (accessed October 1, 2022).

[24] Pinot J Rigal L Granger B Sidorkiewicz S Jaury P. Tailored-dose baclofen in the management of alcoholism: a retrospective study of 144 outpatients followed for three years in a French general practice. Front Psychiatry. (2018) 9:486. 10.3389/fpsyt.2018.00486 30349490 PMC6186794

[25] Garbutt JC Kampov-Polevoy AB Pedersen C Stansbury M Jordan R Willing L. Gallop RJ. Efficacy and tolerability of baclofen in a US community population with alcohol use disorder: a dose-response, randomized, controlled trial. Neuropsychopharmacology. (2021) 46:2250–6. 10.1038/s41386-021-01055-w 34155332 PMC8580979

[26] Dore GM Lo K Juckes L Bezyan S Latt N. Clinical experience with baclofen in the management of alcohol-dependent patients with psychiatric comorbidity: a selected case series. Alcohol Alcohol. (2011) 46:714–20. 10.1093/alcalc/agr131 21890485

[27] Pastor A Jones DM Currie J. High-dose baclofen for treatment-resistant alcohol dependence. J Clin Psychopharmacol. (2012) 32:266–8. 10.1097/JCP.0b013e31824929b2 22367662

[28] Barrault C Lison H Roudot-Thoraval F Garioud A Costentin C Béhar V. One year of baclofen in 100 patients with or without cirrhosis: a French real-life experience. Eur J Gastroenterol Hepatol. (2017) 29:1155–60. 10.1097/MEG.0000000000000922 28727631

[29] Heydtmann M Macdonald B Lewsey J Masson N Cunningham L Irnazarow A. Tailored dose baclofen in patients with alcoholic liver disease: a case series with 2-year follow-up of hospitalization. Addict Res Theory. (2015) 23:510–7. 10.3109/16066359.2015.1040003

[30] Rolland B Labreuche J Duhamel A Deheul S Gautier S Auffret M. Baclofen for alcohol dependence: Relationships between baclofen and alcohol dosing and the occurrence of major sedation. Eur Neuropsychopharmacol. (2015) 25:1631-6. 10.1016/j.euroneuro.2015.05.008 26095229

